# The relationship between house height and mosquito house entry: an experimental study in rural Gambia

**DOI:** 10.1098/rsif.2021.0256

**Published:** 2021-05-26

**Authors:** Majo Carrasco-Tenezaca, Musa Jawara, Mahamed Y. Abdi, John Bradley, Otis Sloan Brittain, Sainey Ceesay, Umberto D'Alessandro, David Jeffries, Margaret Pinder, Hannah Wood, Jakob B. Knudsen, Steve W. Lindsay

**Affiliations:** ^1^ Department of Biosciences, Durham University, Durham, UK; ^2^ Medical Research Council Unit The Gambia at the London School of Hygiene & Tropical Medicine, Banjul, The Gambia; ^3^ Royal Danish Academy - Architecture, Design and Conservation, Copenhagen, Denmark; ^4^ London School of Hygiene & Tropical Medicine, London, UK

**Keywords:** *Anopheles gambiae*, housing, malaria, mosquitoes, sub-Saharan Africa

## Abstract

Most malaria infections in sub-Saharan Africa are acquired indoors, thus finding effective ways of preventing mosquito house entry should reduce transmission. Since most malaria mosquitoes fly less than 1 m from the ground, we tested whether raising buildings off the ground would prevent the entry of *Anopheles gambiae*, the principal African malaria vector, in rural Gambia. Nightly collections of mosquitoes were made using light traps from four inhabited experimental huts, each of which could be moved up or down. Mosquito house entry declined with increasing height, with a hut at 3 m reducing *An. gambiae* house entry by 84% when compared with huts on the ground. A propensity for malaria vectors to fly close to the ground and reduced levels of carbon dioxide, a major mosquito attractant, in elevated huts, may explain our findings. Raised buildings may help reduce malaria transmission in Africa.

## 1. Introduction

The United Nations has projected that the population of sub-Saharan Africa will more than double between 2019 and 2050, and the region will become the world's most populated by 2062 [1]. Coincident with the increasing growth rate, there has been an unprecedented improvement in the housing stock in sub-Saharan Africa, with the proportion of improved houses increasing from 11% in 2000 to 23% in 2015 [2]. With an additional 1.05 billion people in 2050 [1], there has never been a better time to improve the quality of housing in sub-Saharan Africa and make houses healthier for people.

In 2019, there were 384 000 deaths from malaria in sub-Saharan Africa, representing 94% of the global total [3]. It is particularly concerning that the decline in malaria cases has stalled recently and has reversed in some countries despite the massive deployment of insecticide-treated nets (ITNs), indoor residual spraying and prompt and effective treatment with antimalarials. It is generally recognized that supplementary measures are needed to further decrease malaria in the region. Since 79% of bites by malaria mosquitoes occur indoors at night [4], reducing mosquito house entry could contribute to greater malaria control.

There is growing evidence that house design can decrease the force of malaria infection. A systematic review and meta-analysis showed that residents of modern homes had 47% lower odds of malaria infection when compared with traditional homes and a 54–65% lower incidence of clinical malaria [5]. Similarly, a meta-analysis of 29 malaria surveys carried out in 21 sub-Saharan African countries between 2008 and 2015 found that modern housing was associated with a 9–14% reduction in the odds of malaria infection when compared with traditional housing, a level of protection comparable to ITNs in the same study [6]. Modern houses are more likely to have closed eaves (the gap between the top of the wall and the overhanging roof), and screened windows and doors, all modifications known to reduce the entry of malaria mosquitoes into houses [7]. Completely closing a building in the hot humid tropics, however, will reduce ventilation and increase the temperature of the house before midnight, particularly if the roof is metal [8,9]. Making a house hotter at night is bad for malaria control since being too hot is the main reason people give for not sleeping under a mosquito net at night [10]. Thus, the ideal house keeps out malaria vectors, while keeping the occupants cool at night.

We hypothesized that raising a house above the ground would reduce mosquito entry and keep the house cooler. Support for this hypothesis comes from several sources. Firstly, field studies in The Gambia showed that 80% of mosquitoes fly less than 1 m from the ground [11,12]. Secondly, raised platforms can reduce biting by malaria mosquitoes [13,14]. Thirdly, a pilot study in Tanzania showed that screened double-storey buildings had 96% fewer malaria mosquitoes when compared with outdoors levels, while screened single-storey buildings raised less than 1 m from the ground had 77% fewer [15].

**Figure 1.**
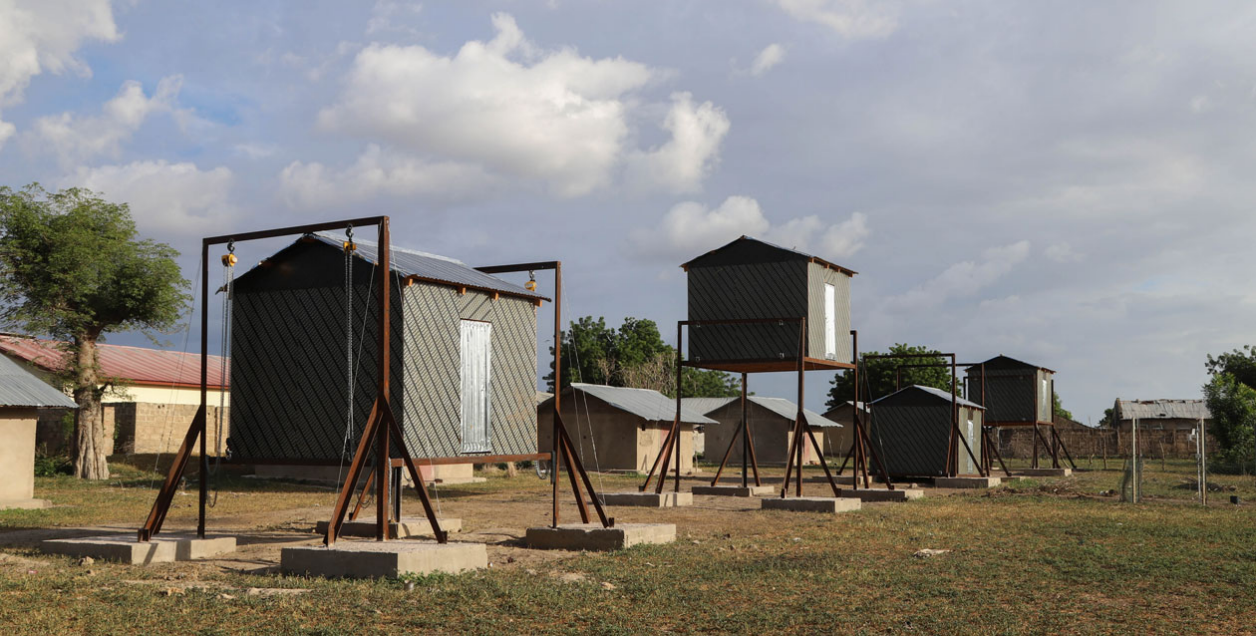
Experimental huts. From left to right, at 1 m, 3 m, 0 m and 2 m. Automatic weather station is shown to the right of the huts and the village to the left and rear.

We used four experimental huts (figure 1), each of which could be adjusted to different heights, to measure house entry of *Anopheles gambiae* s.l., the principal malaria mosquito in sub-Saharan Africa. There were two primary objectives to (i) determine whether mosquito-hut entry declined with increasing height and (ii) find out whether an elevated hut would be cooler than one closer to the ground. This experiment is, to our knowledge, the first study to determine the impact of the height of a house on the entry of malaria mosquitoes and on indoor temperatures at night.

## 2. Material and methods

This was a proof-of-principle study in which four identical huts were built so that each could be moved up or down. All huts had open eaves, no windows and doors with 20 mm gaps at the top and bottom of each to replicate badly fitting doors, common in the region. For four nights each week, one hut was at 0 m, one at 1 m, one at 2 m and one at 3 m (electronic supplementary material, figure S1). The height in which each hut was positioned was changed weekly, following a replicated Latin rectangle design (electronic supplementary material, table S1). Four huts were used instead of one since there is considerable variation in mosquito numbers from night-to-night, and to complete the study within one rainy season.

### 2.1. Study area

The study took place in Wellingara village (N 13°33.365′, W 14° 55.461′), Central River Region, The Gambia. This is an area of flat Sudanese savanna located close to a large area of irrigated rice fields. The study took place in 2019 during the rainy season, from 5 August to 17 October, when *An. gambiae* s.l. are common [9].

### 2.2. Experimental huts

Four experimental huts were constructed, 10 m apart, along a straight line on the western edge of the village, closest to a 1500 ha irrigated rice field. Their design was similar to single-room, one-storey dwellings in the area with doors on opposite sides, but the experimental huts were smaller in size and constructed from lightweight material so they could be easily and safely moved up and down (doi:10.6084/m9.figshare.14483475). The walls of the huts were constructed from 18 mm waterproof plywood on a timber frame and had 0.30 mm tin corrugated roofs (electronic supplementary material, figure S1). Each hut was 3.10 m by 3.10 m in area and the walls were 2.20 m high. Each hut had two corrugated aluminium–steel doors, 1.80 m high and 0.70 m wide, with 20 mm narrow slits on the top and bottom of each door, to simulate badly fitting doors common in villages. One door faced west, towards the rice fields, and the other east, towards the village. The saddle roof was constructed from aluminium–steel sheets mounted on a timber frame, with open eaves on the sides of the house with the doors. Each house had two wooden beds placed parallel to one another on opposite walls, on the sides without doors (north and south). The huts were fixed to a steel frame, which allowed the hut to be raised and lowered using pulley lifts (Yale lever hoist, VSIII 2000 kg Manual Chainblock, Yale, UK). The hoists were anchored in a steel frame that was fixed at ground level by steel foot brackets cast into reinforced concrete foundations (1.65 m × 1.67 m × 0.50 m high).

### 2.3. Study participants

A village meeting was organized to explain the study to the villagers in Mandinka, their main language. Eight healthy men over 15 years old living in Wellingara village provided signed-witnessed consent and were recruited to the study. Before the first experimental session, volunteers slept in the huts for one night to prime the huts with the smell of humans. During the experiments, volunteers slept in the huts for four nights every week for 10 weeks. Each night, each pair of volunteers slept with their heads pointing westward in the direction of the ricefields, under separate ITNs (Olyset Net, 1.30 m wide, 1.50 m high and 1.80 m in length, Sumitomo Chemicals, Japan) from 21.00 to 07.00 the following morning (electronic supplementary material, figure S2). Each pair of volunteers was rotated between huts each night so that at the end of each weekly block, each pair had slept in each hut. Two field assistants were stationed on-site each night in a separate room to assist the volunteers with ladders if they needed to leave the hut during the night.

### 2.4. Outcomes

The main entomological outcomes were the mean number of *An. gambiae* s.l. and the mean temperature of huts. All results were collected at different hut heights before and after midnight. Mosquitoes were collected using one light trap (CDC—Centers for Disease Control and Prevention, miniature light trap model 512, US John W. Hock Ltd, Gainsville, USA) in each hut, with the light bulb 1 m above the floor and between the feet end of the two beds. Light traps were operated from 21.00 to 07.00 the following morning. Every night, the field assistants conducted two supervisory visits, at 00.00 and 06.00, to make sure the men were in the huts, assess bed net use and make sure the light traps were working properly. After collection, mosquitoes were placed in a −20°C freezer until dead. Mosquitoes were identified using standard morphological identification keys [16,17], and members of the *An. gambiae* complex identified to species by PCR [18–20].

Indoor temperature and relative humidity were measured in each hut every 30 min using a data logger (TGU 4500, Tinytag, UK) positioned in the centre of the room, 1 m above the floor. Carbon dioxide was recorded every 30 s with data loggers (1% CO2 + Rh/T Data Logger GasLab, Florida, USA) located between the beds near the head of the bed, 1 m above the floor (electronic supplementary material, figure S2). Outdoor temperature, relative humidity, wind speed, wind direction and precipitation were recorded every 30 min by an automatic weather station (MiniMet, Skye Instruments, Llandrindod Wells, UK), located 10 m from the centre of the huts' line.

At the end of the collection period, all sleepers participated in one focus group discussion. The discussion was led by M.J. and recorded by M.C.T. The men discussed their individual experiences of sleeping in the huts and their willingness to live in a house raised from the ground. After that, they elaborated on common ideas and perceptions of the experimental huts and pointed out at what height they felt more comfortable and explained why. The discussions were conducted in Mandinka, the local language, and audio recorded. The recording was later transcribed into English. Common experiences of the sleepers were reported as quotes for illustration.

### 2.5. Statistical analysis

IBM SPSS Statistics 20 and Stata version 16 were used for the analysis. The sample size was estimated using a computer simulation based on data from a study conducted in the same area in 2017 [9], in which the mean number of *An. gambiae* s.l. collected indoors over 25 nights was 6.4 mosquitoes (SD 7.1). The present study was thus powered to detect an intervention that reduces the number of mosquitoes that were found indoors by at least 75% at the 5% level of significance and 90% power. In the simulation, the 4 × 4 Latin square was repeated three to 10 times (i.e. 12 to 40 nights). The simulation showed that eight, 4 × 4 Latin squares would provide sufficient power to detect a 75% reduction in mosquito house entry (i.e. 32 nights of collections). In this study, we extended the period for two more weeks, because of low mosquito catches in the first two weeks.

To assess the effect of hut height on mosquito house entry and indoor climate, we used a generalized estimating equation using a negative binomial model with a log link function for mosquito count data and a normal distribution with identity link for temperature and carbon dioxide, since they were continuous variables and normally distributed. In addition to hut height, we included hut position, sleeper pair and number of nights in the model as fixed effects. To examine the relationship between carbon dioxide concentration and covariates, we used linear regression. Polar plots were used to depict the direction and strength of the wind during the day and night.

## 3. Results

### 3.1. Mosquito collections

**Figure 2.**
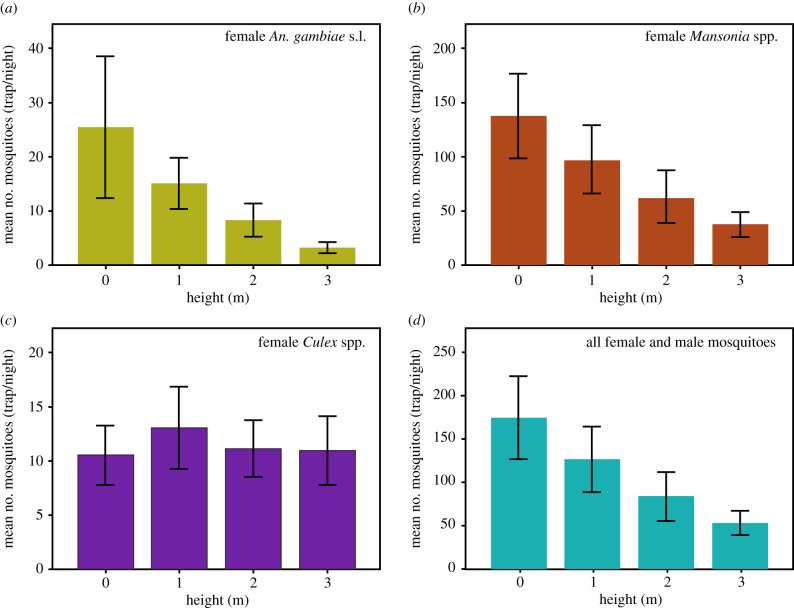
Mean mosquito house entry in huts at different heights. Error bars are 95% confidence intervals. (*a*) *An. gambiae* s.l., (*b*) *Mansonia* spp., (*c*) *Culex quinquefasciatus* and (*d*) all mosquitoes.

A total of 17 432 female mosquitoes were collected in the experimental huts over 40 nights (electronic supplementary material, table S2). Of these, 2080 (11.9%) were *An. gambiae* s.l., 13 321 (76.4%) *Mansonia* spp., 1823 (10.5%) *Culex* spp. and the rest were other anophelines and *Aedes aegypti* (electronic supplementary material, table S2). Members of the *An. gambiae* complex of mosquitoes were identified by PCR analysis as *An. coluzzii* (68.0%, 157/231), *An. arabiensis* (29.4%, 68/231), and *An. gambiae* s.s. (2.6%, 6/231). Overall, unadjusted numbers of mosquitoes of all species entering huts decreased with increasing height (figure 2): by 33% at 1 m, 57% at 2 m and 69% at 3 m, compared with the hut on the ground.

The number of female *An. gambiae* entering the huts declined with increasing hut height, declining by 40% (95% CI 24 to 53%) with the floor height at 1 m, 68% (95% CI 60 to 74%) at 2 m and 84% (95% CI 77 to 88%) at 3 m when compared with the hut at 0 m (table 1). Similar reductions were seen with *Mansonia* spp., but not with *Culex* spp*.*, for which the number of hut-entering mosquitoes was similar in all huts when compared with the hut at 0 m. Details of the regression analyses for *An. gambiae* s.l., indoor temperature and carbon dioxide concentrations are provided in the electronic supplementary material, S3.

**Table 1.** Female mosquitoes collected at different heights and adjusted analysis for covariates. General linearized modelling results, adjusted for house position, sleeper pair and night. CI = confidence intervals.

height of hut (m)	total	mean ratio (95% CI)	effect estimate (95% CI)	*p-*value
*Anopheles gambiae*				
0	1015	reference	—	—
1	601	0.60 (0.47 to 0.76)	−40% (24 to 53)	<0.001
2	333	0.32 (0.26 to 0.40)	−68% (60 to 74)	<0.001
3	131	0.16 (0.12 to 0.23)	−84% (77 to 89)	<0.001
*Mansonia* spp.				
0	5475	reference	—	—
1	3880	0.62 (0.50 to 0.77)	−38% (23 to 50)	<0.001
2	2486	0.35 (0.29 to 0.43)	−65% (57 to 71)	<0.001
3	1471	0.24 (0.18 to 0.30)	−76% (70 to 82)	<0.001
*Culex* spp.				
0	420	reference	—	—
1	522	1.11 (0.79 to 1.58)	+11% (−59 to 21)	0.546
2	444	1.13 (0.95 to 1.35)	+13% (−35 to 5)	0.168
3	437	1.00 (0.86 to 1.15)	0% (−15 to 14)	0.974
all mosquitoes				
0	6998	reference	—	—
1	5069	0.67 (0.43 to 1.06)	−33% (−6 to 57)	0.087
2	3326	0.43 (0.34 to 0.55)	−57% (45 to 66)	<0.001
3	2140	0.31 (0.26 to 0.37)	−69% (63 to 74)	<0.001

### 3.2. Climate measurements

**Figure 3.**
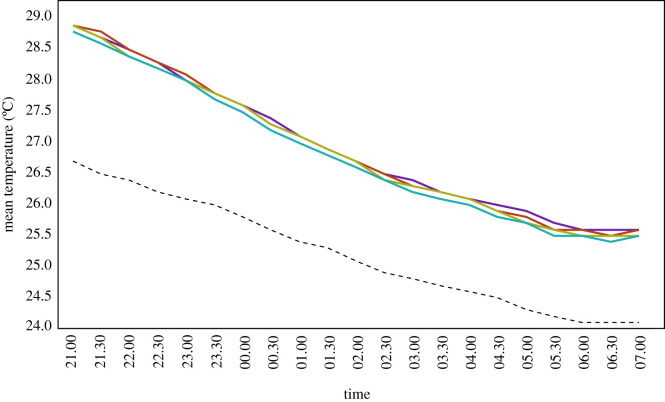
Mean indoor and outdoor temperatures from 21.00 to 07.00. Where purple line = hut at 0 m, red line = hut at 1 m, green line = hut at 2 m, turquoise line = hut at 3 m and dashed black line = outside temperature.

Indoor temperatures declined steadily from 29.0°C at 21.00 to 25.5°C at 07.00, but were always about 2°C warmer than the outdoor temperature (figure 3).

There was little indication that temperature declined with increasing hut height (table 2), although the indoor temperature in the hut at 3 m was of borderline significance when compared with the hut at 0 m, in the period after midnight. No differences in relative humidity were found between huts (electronic supplementary material, table S4). The wind was predominantly from the west, and the mean wind speed was 0.60 m s^−1^ (95% CIs 54.0 to 67.1) from 20.00 to 23.59 and 0.71 m s^−1^ (95% CIs 65.0 to 75.1) from 00.00 to 06.59 (electronic supplementary material, figure S3).

**Table 2.** Environmental measurements outdoors and indoors. General linearized modelling results, adjusted for house position, sleeper pair and night. CI = confidence intervals.

hut height (m)	21.00 to 23.30			00.00 to 07.00		
	mean (95% CI)	difference from reference hut (95% CI)	*p-*value	mean (95% CI)	difference from reference hut (95% CI)	*p-*value
outdoor temperature (°C)						
—	26.5 (26.3 to 26.7)	—	—	24.8 (24.7 to 24.9)	—	—
indoor temperature (°C)						
0	28.4 (28.1 to 28.6)	reference	—	26.4 (26.2 to 26.5)	reference	—
1	28.4 (28.1 to 28.7)	1.05 (0.99 to 1.12)	0.103	26.6 (26.2 to 26. 8)	1.11 (0.90 to 1.37)	0.328
2	28.3 (28.1 to 28.6)	0.98 (0.93 to 1.03)	0.423	26.3 (26.2 to 26.4)	0.95 (0.83 to 1.10)	0.507
3	28.3 (28.0 to 28.5)	0.97 (0.91 to 1.02)	0.211	26.2 (26.1 to 26.4)	0.88 (0.76 to 1.01)	0.067
indoor carbon dioxide levels (ppm)						
0	760 (710 to 800)	reference	—	710 (680 to 750)	reference	—
1	710 (680 to 740)	75.2 (60.6 to 93.4)	0.010	680 (650 to 710)	80.3 (70.1 to 92.0)	0.002
2	710 (680 to 740)	60.2 (50.7 to 71.5)	<0.001	670 (640 to 700)	74.6 (64.9 to 85.9)	<0.001
3	690 (660 to 730)	55.2 (45.9 to 66.4)	<0.001	660 (630 to 700)	61.6 (53.9 to 70.5)	<0.001

### 3.3. Carbon dioxide measurements

**Figure 4.**
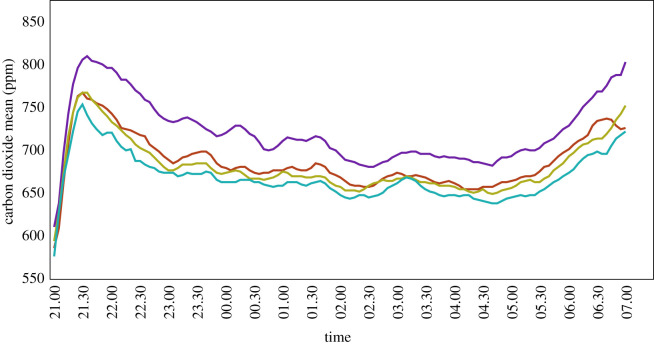
Mean carbon dioxide concentration from 21.00 to 07.00. Where purple line = hut at 0 m, red line = hut at 1 m, green line = hut at 2 m and turquoise line = hut at 3 m.

Although there was considerable nightly variation in the pattern of carbon dioxide in each hut, the mean values provide consistent patterns and show clear trends between huts (figure 4). Carbon dioxide levels indoors rose sharply after the men entered the huts to a maximum value 30 min later. Thereafter, levels gradually declined during the night before rising sharply around 05.00 to a second, smaller peak at 07.00, when the men left the huts.

There was a decreasing trend in the concentration of carbon dioxide with increasing height, both in the periods before and after midnight (table 2). Before midnight, there was a 24.8% decline in carbon dioxide at 1 m, 39.8% at 2 m (rate ratio, RR = 0.60, 95% CI = 0.51 to 0.72) and 44.8% at 3 m (RR = 0.55, 95% CI = 0.46 to 0.66) when compared with the hut at 0 m (electronic supplementary material, table S4). While after midnight, there was a 19.7% decline in carbon dioxide at 1 m, 25.4% at 2 m (RR = 0.75, 95% CI = 0.65 to 0.86) and 38.4% at 3 m (RR = 0.62, 95% CI = 0.54 to 0.71) when compared with the hut at 0 m.

### 3.4. Focus group discussion

A complete transcript of the focus group discussion with the men who slept in the experimental huts is provided in FigShare (doi:10.6084/m9.figshare.14483475).

Briefly, sleepers preferred to sleep in the huts at 0 m and 3 m. When asked the reasons for their choice, they mentioned that the higher hut was cooler and had fewer mosquitoes disturbing them during the night. The sleepers also said they did notice a change in temperature throughout the night, with temperatures becoming cold after midnight.

‘*I am very happy whenever I am going to level three* [3 m hut]. *I am very, very happy*.’

‘*Initially is very, very hot inside, before twelve. By twelve it starts to get cold*.’

Sleepers said that they would live in a house raised from the ground if it was made of solid (mud block) materials like traditional houses and had a fixed stair to access the house if it was to be permanent.

‘[Huts would be more useful] *if the houses are solid, like the way the ones* [built] *on the ground. We would prefer that*.’

‘*As I said, I prefer the stair that is fixed to the house. I prefer if all the stairs would be in that form. It would be much more appreciated*.’

### 3.5. Serious adverse events

There was one serious adverse event during the study, which occurred when one study participant sleep-walked out of the 2 m high hut and received minor injuries. This accident was most likely to have been caused by the medication the participant was taking, and he was replaced with a healthy volunteer. Hut doors were secured during the night to prevent any further accidents.

## 4. Discussion

Our findings establish how house height affects host location by the world's most efficient vector of malaria and provides a plausible mechanism for this behaviour. The number of female *An. gambiae* mosquitoes collected in the huts declined with increasing height, decreasing progressively with increasing height of the hut's floor above the ground. Huts with the floor 3 m above the ground had 84% fewer mosquitoes than those on the ground [21]. If this reduction correlates to a similar reduction in malaria transmission, it would be comparable to that of an ITN that can reduce malaria transmission by 40–90% [21]. Similar reductions in *Mansonia* spp., vectors of lymphatic filariasis and arboviruses, were observed as the height of a house was raised, with the huts at 3 m having 77% fewer mosquitoes than the hut at 0 m. In marked contrast, the number of *Culex* spp. entering experimental huts was similar at any height.

Our findings are supported by a series of studies that measured the height at which mosquitoes fly which were conducted in the same study area [11] and two other sites in The Gambia between 1968 and 1977 [12,22]. Mosquitoes were collected at different heights using suction traps mounted on scaffolding, with 80% of the total catch of mosquitoes collected less than 1 m from the ground [22]. The researchers differentiated between low-flying mosquitoes, like *Anopheles* spp*.* and *Mansonia* spp*.*, *Aedes punctothoracis* and higher-flying mosquitoes that could be collected at 3.5 to 4 m, which included *Culex neavei* and *Cx. weschei*. In The Gambia, many *Culex* species, like *Cx. neavei*, feed primarily on birds [23] and, presumably, the ability of these mosquitoes to fly at higher altitudes, when compared with most mosquitoes, allows them to feed more readily on birds roosting at night in trees. Although *An. gambiae* s.l. and *Mansonia* spp. normally fly close to the ground, when they reach a house they fly upwards and typically enter through open eaves, while mosquitoes like *Aedes* spp., *An. pharoensis*, *Cx. poicilipes* and *Cx. thalassius* do not [12,24]. In a study conducted in the present study area, it was found that *An. gambiae* and *Mansonia* spp. moved up over a 6 m high circular netting fence to feed on people inside the fence [11]. This study raises the question of whether house height is protective if houses raised on stilts are closed underneath with netting or other materials. Reassuringly, a study in Tanzania found that mosquito entry decreased in two-storey houses when compared with one-storey houses [15]. In Kenya, thatched-roofed houses with mud walls and raised on stilts had 81% fewer malaria vectors than houses built from the same material but on the ground [25]. Similarly, in São Tomé, houses on stilts had half the number of *An. gambiae* s.l. than those built on the ground [26]. These observations could be due to the position of the house, rather than the house height. In our study, however, because we adjusted for house position in the design, we can be confident that house entry of *An. gambiae* s.l. declines with house height.

In the current experiments, temperature declined progressively through the night. There was no consistent evidence that indoor temperature declined with the height of the hut. This was unexpected since it was hypothesized that air flowing underneath the raised huts would decrease their indoor temperature when compared with the hut located on the ground, and at higher altitudes, where wind speed increases [27]. There are two likely explanations for our findings. (i) The huts in our study were built with materials of low thermal mass that lose heat rapidly. If the buildings were made of mud, concrete or brick, high thermal mass materials, we may have found a difference in cooling with increased height. (ii) The hut at 0 m had a gap of 500 mm between the ground and wooden floor. The air beneath the hut acts as an insulating layer and is likely to make the hut cooler than if it had been built directly on the ground. By contrast, in Tanzania, two-storey houses were 2.3°C (95% CI 2.2 to 2.4) cooler than single-storey houses [15].

Carbon dioxide levels rose rapidly after the study participants entered the huts in the evening and then slowly declined through the night until rising around 05.00 to a second peak at 07.00, when the men left the huts. Both peaks were partly associated with increased physical activity, the first, chatting and preparing to go to bed, and the second, praying and getting ready to leave the huts in the morning. The second peak is also likely to be under circadian control, representing an increase in metabolic activity prior to awakening [28]. Similar upticks of activity are seen with an increase in core temperature before wakening [28,29]. The prolonged decline in carbon dioxide during the night is associated with a decline in physical activity and sleep, with an associated reduction in respiratory rate and increased carbon dioxide concentrations in the body [30]. Carbon dioxide levels declined as the height of the hut increased, most probably due to stronger winds at higher elevations [27]. Since carbon dioxide and other volatiles produced by humans are strong attractants for blood-questing mosquitoes [31], the lower concentrations experienced in elevated huts are likely to contribute partly to the decline in mosquitoes seen with increasing hut height. Carbon dioxide concentrations and human-produced volatiles emanating from a house are key to understanding our findings and probably explains why houses with more people have more mosquitoes than those with few or none [32].

During the focus group discussion, participants stated a preference to sleep in huts at 0 m and 3 m. It is likely that their preference for the hut at ground level was associated with the familiar style of housing in this community, all of which are single-storey ground-level buildings. This conclusion was supported by a preference for ‘solid’ materials. Hence, there is a perceived ‘norm’ for local houses to be built on the ground and of solid materials. Nonetheless, participants did appreciate the hut at 3 m because of the absence of mosquitoes. The huts were designed to answer a particular hypothesis, and they were not intended to represent what actual houses would look like in the future. These would be robustly constructed with lightweight materials, well ventilated and with safe stairs for children and adults [15].

**Figure 5.**
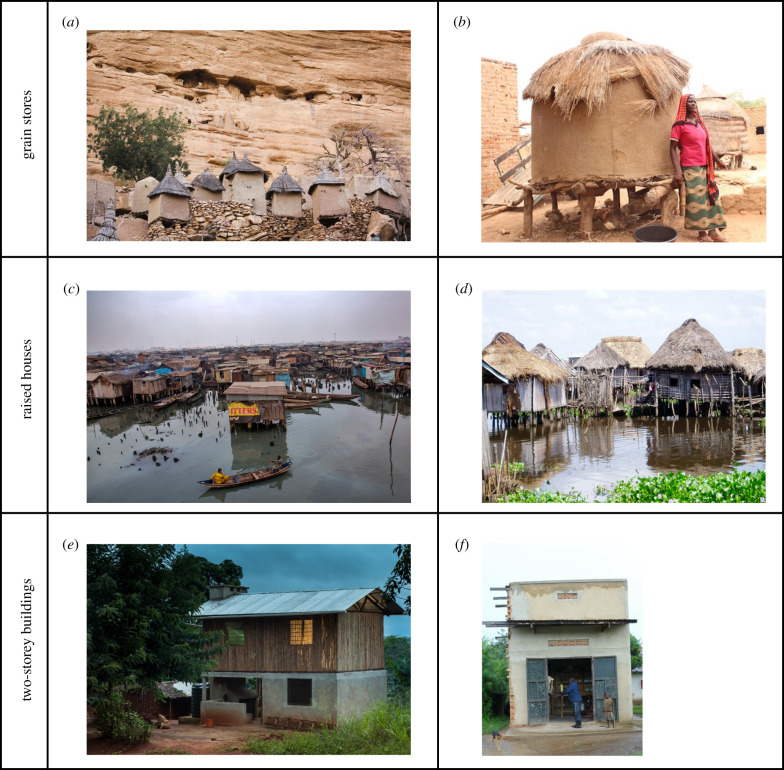
Raised and two-storey constructions in sub-Saharan Africa. (*a*) Dogon granaries in Mali, 2016 (Hamaji Magazine); (*b*) granaries for crop storage in Chad, 2017 (UN Environment Programme); (*c*) Makoko in Nigeria, 2016 (The Guardian); (*d*) Ganvie in Benin, 2018 (Scribol Magazine); (*e*) double-storey bamboo prototype house in Tanzania [15]; (*f*) two-storey house with store in the ground floor in Uganda (S.W.L.).

With the population of sub-Saharan Africa expected to increase sharply over the next decades [1], there is a need to produce millions of healthy, comfortable, safe and cheap houses. While most rural houses in the region are single-storey buildings, constructed on the ground [33], there are numerous examples of indigenous structures built off the ground (figure 5). Raised structures are built for four reasons. Firstly, as grain stores, elevated to prevent infestations with rodents, such as the Kongo Granaries in Angola [34] and multi-storey Dogon granaries in Mali [35]. Secondly, houses are built on stilts to avoid damp or flooded ground, such as those constructed by the Lafofa people in southern Sudan [35], the lake village of Ganvie, in Benin, a UNESCO World Heritage site [36,37], and Makoko, one of Lagos' largest informal settlements [38], with a third of its area built over a lake. Such structures are typically built on the coast or riverside as protection against flooding [35]. Clearly, installing sturdy two-storey buildings in areas prone to flooding is highly desirable, particularly as flooding becomes more common due to climate change [39]. Thirdly, they may be used as a defence and in the past were used as protection from slave-raiding parties [40]. Fourthly, today two-storey houses or higher are common because of a shortage of land, in order to increase living space and have a more efficient use of land [41,42]. These structures are part of the urban landscape of many African cities and towns, and alongside busy roads where shops are frequently two-storey structures. As land becomes in increasingly short supply, the building of multi-storey infrastructure will undoubtedly increase.

There are several limitations to our study. Firstly, the experimental huts were smaller than traditional single-room houses and made with marine plywood walls instead of mud or cement blocks. This made them hotter than traditional houses during the day, and the movement of carbon dioxide in these buildings will differ from typical houses. Secondly, in the study area, adults usually go to bed 2–3 h later [43] than in our study. Thirdly, we do not know whether closing the space under an elevated house will result in more mosquitoes flying up the sides of the building and entering the dwelling room in higher numbers than a similar building that is open underneath. Studies in Tanzania, however, indicate that this will not reduce protection from mosquitoes [15]. Fourthly, with our current design, we were unable to determine exit rates of mosquitoes leaving the huts or measure the number of mosquitoes that died during the night. Finally, our study was conducted in experimental huts, which means that the performance of the house and perceptions from possible users will differ when compared with implementation or scaling up a prototype.

Our findings show that the number of malaria mosquitoes entering a hut declines with the increasing height of the hut. This results from the habit of most mosquitoes flying less than 1 m above the ground and lower production of carbon dioxide and other attractants in the cooler elevated houses. Essentially, mosquitoes are less likely to feed on a person sleeping in an elevated house than one on the ground. At 3 m, this reduction in an indoor entry is equivalent to the protections afforded by an insecticide-treated bed net [21]. Raising houses off the ground, like any intervention, is not evolutionary proof, and over time, mosquitoes may adapt and feed higher off the ground than before. Nonetheless, we recommend elevating houses off the ground since they are likely to reduce mosquito biting and keep the occupants cooler at night [7] and therefore more likely to use an ITN. This research is likely to be relevant to many hot and humid parts of sub-Saharan Africa where *An. gambiae* s.l. is the major vector of malaria and places where high temperatures reduce the use of bed nets. Raising houses off the ground is likely to reduce mosquito house entry.

## Data accessibility

Data files and video are available on FigShare (doi:10.6084/m9.figshare.14483475) [44].
